# Personality Traits and Behavioral Syndromes in Differently Urbanized Populations of House Sparrows (*Passer domesticus*)

**DOI:** 10.1371/journal.pone.0036639

**Published:** 2012-05-04

**Authors:** Veronika Bókony, Anna Kulcsár, Zoltán Tóth, András Liker

**Affiliations:** 1 Department of Limnology, University of Pannonia, Veszprém, Hungary; 2 Department of Physiology and Biochemistry, Faculty of Veterinary Sciences, Szent István University, István, Budapest, Hungary; 3 Institute of Environmental Sciences, Jagiellonian University, Krakow, Poland; Arizona State University, United States of America

## Abstract

Urbanization creates novel environments for wild animals where selection pressures may differ drastically from those in natural habitats. Adaptation to urban life involves changes in various traits, including behavior. Behavioral traits often vary consistently among individuals, and these so-called personality traits can be correlated with each other, forming behavioral syndromes. Despite their adaptive significance and potential to act as constraints, little is known about the role of animal personality and behavioral syndromes in animals' adaptation to urban habitats. In this study we tested whether differently urbanized habitats select for different personalities and behavioral syndromes by altering the population mean, inter-individual variability, and correlations of personality traits. We captured house sparrows (*Passer domesticus*) from four different populations along the gradient of urbanization and assessed their behavior in standardized test situations. We found individual consistency in neophobia, risk taking, and activity, constituting three personality axes. On the one hand, urbanization did not consistently affect the mean and variance of these traits, although there were significant differences between some of the populations in food neophobia and risk taking (both in means and variances). On the other hand, both urban and rural birds exhibited a behavioral syndrome including object neophobia, risk taking and activity, whereas food neophobia was part of the syndrome only in rural birds. These results indicate that there are population differences in certain aspects of personality in house sparrows, some of which may be related to habitat urbanization. Our findings suggest that urbanization and/or other population-level habitat differences may not only influence the expression of personality traits but also alter their inter-individual variability and the relationships among them, changing the structure of behavioral syndromes.

## Introduction

Urbanized areas are expanding and developing at an accelerating rate throughout the world, and this process has profound effects on wildlife [1]–[4]. Animal species differ greatly in the extent to which they can tolerate, invade or persist in urban environments, and these differences have been attributed to several characteristics of the species' ecology, life history, physiology and behavior [5]–[9]. A key predictor of successful urbanization seems to be the reduced fearfulness of humans, as several recent studies have suggested that only the tamest individuals are able to colonize urban habitats [7]–[8], [10]–[11]. Besides human disturbance, however, urbanization exposes animals to a variety of novel opportunities and risks such as novel food sources [12]–[13], alternative breeding sites [14]–[15], and an altered fauna of predators compared to natural habitats [16]. Behavioral adaptations to these challenges may also play a crucial role in successful “city life”.

Behavioral traits often differ among individuals and are expressed consistently over time and across situations; such traits have been termed personality traits or temperament traits [17]–[18]. For example, great tits (*Parus major*) that are more explorative in an unfamiliar environment also approach novel objects more quickly [17], and salamanders (*Ambystoma barbouri*) that are very active during foraging are also overly active in the presence of predators [19]. These personality traits constitute the axes of animal personality, such as responses to novelty, risk taking, and activity (reviewed by [18]). Personality traits can be correlated with each other, for example, more explorative great tits are also more aggressive and ready to take risks [17]; such behavioral correlations have been termed behavioral syndromes [18], [20]. Personality traits can have significant fitness consequences in nature (reviewed by [21]), and the pay-offs of different personalities can vary with environmental conditions in time or space [17]–[18]. For example, bold bighorn sheep (*Ovis canadensis*) survived better than shy ones in years with high predation but not when predation was low [22]. However, personality traits can also act as constraints on optimal behavior [20], for example, being active may be adaptive during foraging but not in the presence of predators [19]. Furthermore, different environments may favor different behavioral syndromes; for example, boldness and aggression were correlated in stickleback (*Gasterosteus aculeatus*) populations that lived sympatrically with predators but not in populations without predators [23]–[25]. Thus, given the diverse effects of urbanization on both abiotic and biotic environmental conditions for animals [2]–[4], it can be expected that selection for personality traits and behavioral syndromes varies along the gradient of urbanization. Moreover, urbanization may also select for higher inter-individual variability of personality traits, because a greater diversity of personalities can exploit a greater diversity of resources and niches [11]. As an extension to this idea, urbanization may also select for a greater variability in the associations between personality traits, which may then change or weaken behavioral syndromes that may be adaptive in natural habitats.

Several studies up to now have found behavioral differences between differently urbanized populations, for example, in responses to novelty and problem solving [26]–[28]; risk taking [29]–[32] and activity [12]. However, very few have investigated such behavioral differences within the framework of personality and behavioral syndromes. To our knowledge, the only research that has demonstrated individual consistency in the behavioral traits compared between urban and rural conspecifics is a series of studies on song sparrows (*Melospiza melodia*), which showed that urban birds are less fearful of humans and more aggressive in territory defense than rural birds [33]–[34]. Very little, if anything, is know about the influence of urbanization on the expression and relationships of other personality traits such as neophobia (i.e. fear of novelty), activity, and fearfulness of non-human predators. Similarly, how urbanization affects the diversity of personalities is largely unknown. Phylogenetic comparative studies of birds indicated that both mean and variance of fearfulness of humans is reduced during the colonization of urban habitats [8], [11] but later, as species become more urbanized, their inter-individual variability in fearfulness increases [11]. However, no consistent habitat difference in inter-individual variability was found in song sparrows' fearfulness of humans and territorial aggression [33]–[34] and in house sparrows' (*Passer domesticus*) aggression and other individually consistent competitive behaviors [35]. Nevertheless, song sparrows' fearfulness of humans and aggression were correlated in rural but not in urban populations [33]–[34], suggesting that the combination of the two traits is more flexible in urban habitats, leading to breakdown of the behavioral syndrome. Clearly, more studies are needed if we are to understand how habitat urbanization influences animal personalities.

In this study, we investigated personality traits and behavioral syndromes in bird populations along the urbanization gradient to test the hypotheses that differently urbanized habitats favor different expression and diversity of personality traits. Out of the five personality axes suggested for non-human animals [18] we focused on three: neophobia as proxy for the exploration-avoidance axis, predatory risk-taking as proxy for the boldness-shyness axis, and the general level of activity. Our study species is the house sparrow, a small passerine bird that has been the subject of many behavioral studies [36], yet the existence and significance of personalities in this species is largely unexplored [37]–[38]. It is a unique model species for studying urbanization, since during its long common history with humans it has occupied a wide variety of habitats from rural farmlands to the most heavily urbanized areas, and kept adapting its behavior to the development of human settlements e.g. by switching from feeding on horse dung on the streets to as tricky methods as fluttering in front of the electronic sensor of automatic doors to get access to restaurant food [36]. Due to the species' sedentary nature, restricted dispersal and small home range, urban and rural populations comprise distinct genetic clusters [39] which may facilitate local adaptations in behavior. Therefore we compared the behavior of individuals from differently urbanized populations in a number of standardized test situations to explore the within-individual consistency, population mean, inter-individual variance, and relationships of their personality traits.

## Methods

### Study subjects

We captured 60 house sparrows with mist nets (Ecotone, Poland) between 1–18 Oct 2007 in four differently urbanized habitats in Hungary (Table 1; see Appendix 1 in [35]). Two more urbanized sites are within the densely built areas of two cities (the capital of Budapest and the town of Veszprém), whereas two less urbanized sites are extensively surrounded by non-built, agricultural areas (at the edge of a village Nemesvámos and at a small, isolated farm Dóramajor). We followed [40] to quantify the degree of urbanization in each habitat by scoring the cover of vegetation, paved roads, and buildings in a 1 km^2^ area around each capture site, and we also collected data on the density of multi-storey buildings and the residential human population for each settlement (Hungarian Central Statistical Office, Population Census 2001, http://www.nepszamlalas.hu/eng/index.html) as suggested by [1]. Then we calculated an “urbanization score” for each site as the PC1 score from a principal component analysis of the seven measures of urbanization (Table 1). The analysis extracted one principal component that accounted for 85.1% of the total variance and correlated negatively with vegetation cover and positively with the density of buildings, roads and humans (Table 1).

**Table 1:** Characteristics of the capture sites and sample sizes.

	Budapest	Veszprém	Nemesvámos	Dóramajor
Capture site	urban train and bus station	urban quick-food restaurant	rural dairy farm	rural horse and cattle breeding farm
Geographical coordinates	47°28′ N, 19°09′ E	47°05′N, 17°55′E	47°03′N, 17°52′E	47°21′N, 19°19′E
Urbanization score*	1.08	0.60	−0.68	−1.00
Mean vegetation density score (−0.99)*	1.03	1.15	1.71	1.97
Number of cells with high vegetation density (−0.99)*	11	19	75	97
Number of cells with road (0.96)*	93	98	27	26
Mean building density score (0.94)*	1.15	1.33	0.49	0.35
Number of cells with high building density (0.98)*	37	37	11	1
Density of multi-storey buildings / km^2^ (0.79)*	75.6	9.3	0	1
Human population density / km^2^ (0.78)*	4524.5	471.5	63.8	49
Number of birds tested (males, females)	20 (10, 10)	9 (6, 3)	18 (7, 11)	11 (3, 8)

* Vegetation cover, building density, and the presence of roads were scored for 100 cells of a 1 km^2^ area around each capture site; the mean of the 100 cell scores are given for each site. Density of multi-storey buildings and residential human population are given for each settlement. Urbanization scores are the PC1 values from a principal component analysis of the seven habitat variables; component loadings are given for each variable in brackets.

Upon capture, we ringed each bird with a numbered aluminium ring and 3 color rings. Sparrows were transported to Veszprém, where they were housed in outdoor aviaries (ca. 3×4 m, 3 m high) in four mixed flocks (14–16 individuals per flock), each containing an equal number of urban and rural birds. We provided roosting trees and small boxes as shelter, *ad libitum* food (millet, wheat, and sunflower seeds) and water supplemented with multivitamin droplets. During the 4-months course of this study, an urban and a rural bird died for unknown reasons; this rate of mortality (3.3%) is small compared to that observed in free-living house sparrows and similar to that observed in other studies of captive sparrows [27], [36]. The rest of the birds remained apparently in good condition and many of them even bred successfully in the aviaries in summer 2008. Capture, housing and handling of birds were in accordance with the relevant Hungarian laws and were licensed by the Balaton Upland National Park (permission number: 9135-2/2004).

### Behavioral tests

We conducted behavioral tests in December 2007 and January 2008 to assess the birds' personality traits (n = 58 birds). The tests were run in 8 one-week long test periods in which 6 or 8 birds were tested simultaneously each week. At the beginning of each test period we captured the half of a flock (choosing randomly) from one of the aviaries, weighed the birds and housed them in individual indoor cages. Each cage (52×46×37 cm) contained a feeder cup, a water pot, a horizontal perch and a shelter box; the shelter and the feeder were placed in the opposite ends of the cage (back and front, respectively). The wire mesh bottom of the cages prevented the birds from accessing spillage, so they could only feed from the feeder. The 8 cages were placed on 4 shelves above each other, isolated visually (but not acoustically) from each other by opaque slides. Birds were allocated in the cages randomly, but each shelf and each column contained equal number of urban and rural sparrows.

After being placed in the cages, birds were left undisturbed for two days (days 1 and 2) with *ad libitum* food (millet and wheat) and water. On the following 5 days (days 3–7), birds participated in a series of behavioral tests in which their behavior was observed each morning from 8:00 to 9:00, and the food was available from 8:00 to 16:00. All tests involved providing food to the birds in the morning after their overnight fast, and observing their behavior for one hour from behind a curtain through a one-way window and also recording by two video cameras for detailed analysis (each camera recorded 4 cages simultaneously). Because birds may gradually habituate to the experimental protocol, we chose to conduct the behavioral tests in a fixed order [28], starting with the simplest task (i.e. a control test) and ending with the most risky situation (i.e. raptor test) to prevent the tests from being overwhelming to the birds. To detect habituation, however, we performed a second control test on the penultimate test day.

The first test, on day 3, was a control test. After starting video-recording, the observer quickly placed the feeders containing the familiar seed mixture into the cages, then hid behind the curtain and observed the behavior of the birds.

In the novel object test on day 4, the above procedure was repeated but a novel object was also placed into the cages next to the feeder. Four different kinds of objects were used: a yellow tennis ball, a silver toy car, a toy Santa Claus, and a colorful paper roll decorated with colorful straws [27], [41]. The size of the objects was similar (7–10 cm) but they differed in color and shape (see below for rationale of using different stimuli). Objects were randomly distributed among cages, with all types used at least once per week, but each type was used for an approximately equal number of birds from each habitat. At the end of the one-hour observation, the experimenter took out the objects and birds were left undisturbed until 16:00.

In the novel food test on day 5, birds got unfamiliar food in the feeder at the start of test instead of the seed mixture. Four different kinds of food were used: kiwi slices, grated hard cheese, colored sweet rice flakes, and cottage cheese with raisins [41]. Novel food types were distributed among cages following the same rules as for novel objects; we varied the types of objects and foods independently of each other among birds. At the end of the one-hour observation, novel food was replaced by familiar food for the rest of the day until 16:00. In both novelty tests, we used four different test stimuli to ensure the generality of our comparisons among populations, i.e. that the neophobia we measured was not merely a specific response to a certain object or food. To this end, we traded off some accuracy in the individual neophobia scores because each individual was tested with only one type of object and food (due to the time-consuming nature of behavioral tests). Despite this source of noise, however, our measures of neophobia still showed individual consistency (see Results).

In the second control test (dove test) on day 6, the procedure of the control test (day 3) was repeated, but for the first 5 minutes of observation a taxidermy-mounted collared dove (*Streptopelia decaocto*) in perching position was placed ca. 1 m in front of the cages where it could be visible to all birds. House sparrows often forage together with collared doves, so this species was chosen as a control for the presence of a dummy for the raptor test (see later). After 5 minutes, the experimenter removed the dummy by reaching out and pulling it behind the curtain, and kept observing and videotaping the birds for the rest of the one-hour recording.

In the raptor test on day 7, the procedure of the dove test was repeated but sparrows were exposed to a dummy sparrowhawk (*Accipiter nisus*) instead of a collared dove. The sparrowhawk is one of the main predators of house sparrows [36]. In 7 out of the 8 test groups, birds responded to the dummy sparrowhawk by alarm calling while it was present. After this test, birds were weighed again and released back to the aviary.

### Data analysis

From the video-recordings of each test, we collected data on the following two variables for each individual. (1) Latency to feed was measured as the time (sec) elapsed from the start of test (i.e. when the experimenter hid) until the bird first pecked from the feeder. If an individual did not approach the feeder or peck from it at all during a test, it was given a latency value of 3720 sec (maximum duration of recordings). (2) From six 60-sec samples chosen randomly from each one-hour recording (one sample in every 10 min), we measured the number of hops as the total number of times the bird hopped from the perch into the front or the back of the cage. This variable was used as an estimate of activity (note that counting hops for the entire 60 min of each test for all birds would have been extremely time-consuming).

Throughout the analyses, we investigated habitat effects by two parallel approaches. On the one hand, we compared urban birds (i.e. from the two more urbanized populations) to rural birds (i.e. from the two less urbanized populations) since the urbanization scores of capture sites supported this urban-rural split-up (Table 1). On the other hand, we also compared the four differently urbanized populations wherever possible because behavioral traits might change in a non-linear fashion along the urban gradient, in which case pooling different populations may mask the effects of urbanization. Note that the latter approach was not feasible with SEM analyses of syndrome structure (see below).

First we compared feeding latencies and number of hops across birds from different habitats (i.e. urban versus rural) or from the four different populations by the following methods. Because feeding latencies were truncated at 60 minutes, we applied survival analyses that can handle such censored data appropriately. Feeding latency in each test was thus analyzed as a function of habitat using Cox proportional hazards models [42]. In the case of the novelty tests and the raptor test, feeding latencies in the respective control tests were also included as covariates. In the models of the novelty tests, we also included the type of novel object or food, respectively, as fixed factors. Initially, we also included the potentially confounding effects of sex, cage position, test group (i.e. birds tested at the same time), and body mass; however, these variables were retained only if their exclusion would have worsen model fit based on the increase in deviance, otherwise they were dropped to obtain a minimal adequate model (MAM) for each dependent variable [42].

The number of hops in the five consecutive tests was analyzed in a generalized linear mixed-effects model with quasi-Poisson error distribution [43]. Test group and bird ID were included as nested random factors, which enabled that values of an individual in different tests were treated as repeated measures and also allowed the values of birds in the same test group to co-vary. The potentially confounding variables of sex, cage position, date, and body mass were dropped from the initial model because their effects were non-significant.

Then we compared the variance of feeding latencies and number of hops across birds from different habitats by the Brown–Forsythe test. This method is similar to Levene's test but uses the deviations from the median instead of the mean, thereby it performs better for skewed distributions [44].

**Figure 1:**
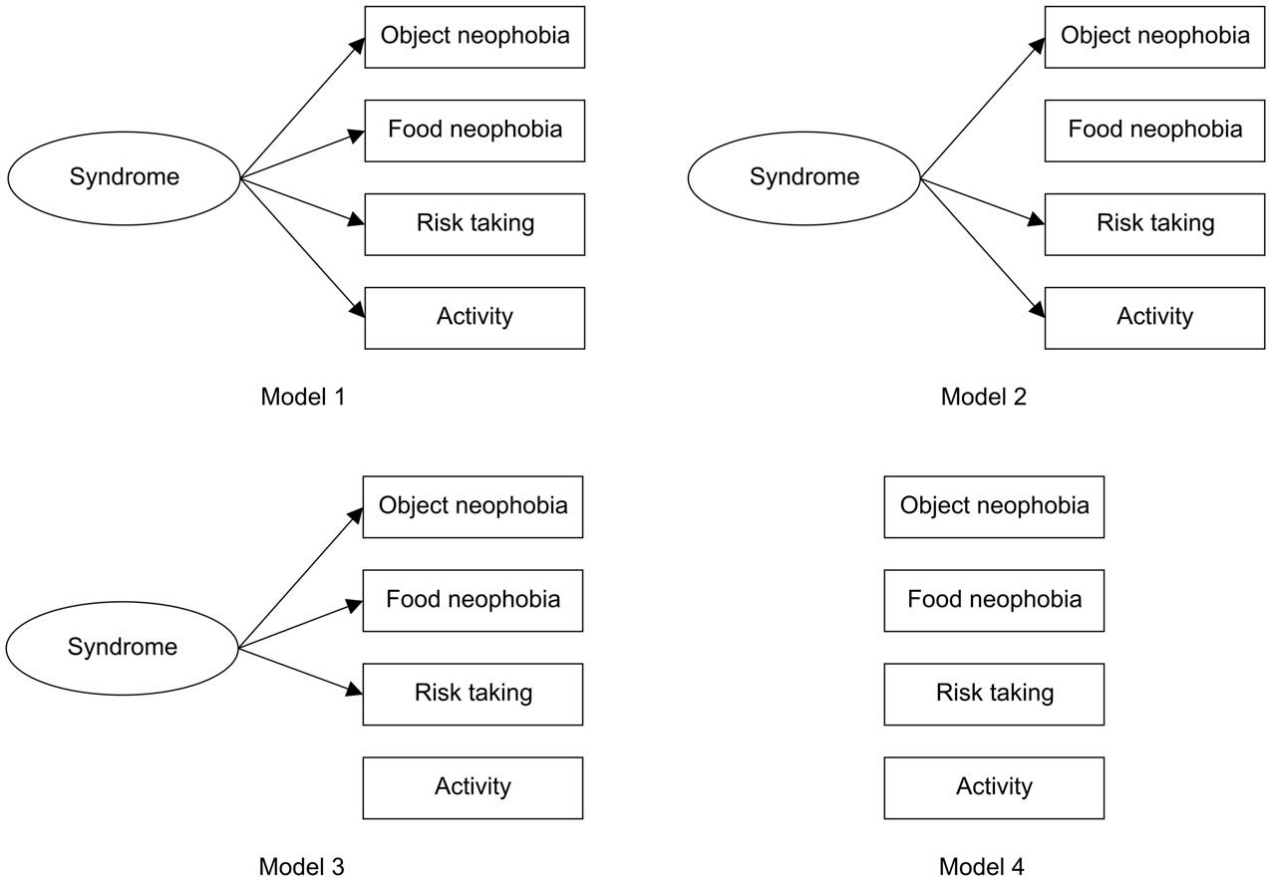
Four candidate models of behavioral syndrome structure. Measured behavioral traits are shown in rectangular boxes; underlying causal effects (latent variables) responsible for syndrome structure are shown in ovals.

To investigate behavioral syndromes, we applied structural equation modelling (SEM) [25], [45] within urban and rural birds, respectively (our sample sizes did not permit SEM analyses for the four populations separately). To keep the number of variables low enough, we restricted the analyses to the four most relevant traits, namely feeding latencies in the novel object test (object neophobia), novel food test (food neophobia) and raptor test (risk taking), and the average number of hops over all tests (general activity). Because we had censored data (i.e. maximal latencies) for both novelty tests, first we performed Bayesian estimation to impute the censored data [45], [46] using a SEM model of syndrome structure that included all four personality traits (Model 1 in Fig. 1) for all birds (n = 58). We used non-informative priors with the constraint that prior density was set to zero for non-positive variances. We estimated the posterior distribution for each censored latency value by Markov chain Monte Carlo (MCMC) simulation, and imputed the posterior mean values to obtain a dataset for further analyses [45]. The imputed variables were then z-transformed to remove differences in mean and variance among the four populations and the four test objects or food types, and to better accomodate the requirement of SEM for multivariate normal distributions [25]. We formulated four *a priori* hypotheses of syndrome structure (Fig. 1) based on our knowledge of behavioral syndromes in general and in house sparrows specifically. Model 1 represents a domain-general model of syndrome structure [20], [25] with object neophobia, food neophobia, risk taking, and activity all linked via a common source depicted by the latent variable „syndrome”. Model 2 represents a similar syndrome structure with more restricted domain-generality that does not include food neophobia, because this behavior can be contextually different from other novelty responses [25], [38]. Model 3 represents a case where behavioral responses to risky and novel stimuli are correlated with each other but not with levels of general activity, given that activity and exploration are not correlated in house sparrows [38]. Model 4 represents the absence of syndrome, with behavioral traits varying independently of each other [25]. We ran a Bayesian SEM analysis [45], [46] for each of these hypotheses, then compared the models using the deviance information criterion (DIC) which is a generalization of Akaike's information criterion (AIC) for Bayesian model selection by MCMC simulation [45].

All statistical analyses except SEM were performed using R 2.7.2 [47] and its following packages: lawstat, nlme, MASS, survival. For SEM we used AMOS 20.0, SPSS Inc [45]. Results are presented as mean ± SE, and all tests are two-tailed with a 5% significance level. In the case of multiple comparisons, significance levels were adjusted by the false discovery rate (FDR) method as suggested by [48].

## Results

### Responses to test situations

**Figure 2:**
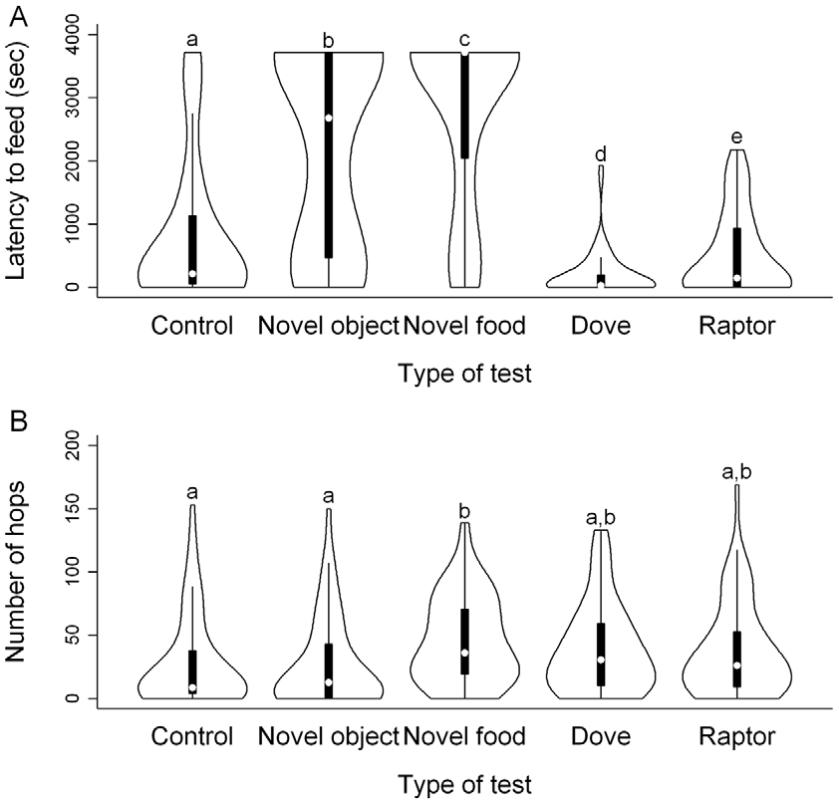
Effects of test type on (A) latency to feed and (B) number of hops. Each violin plot shows the distribution of the data by a box plot (median as a white dot, interquartile range as a black box, data range as whiskers) and a kernel density plot on each side of the box plot. Letters above the plots mark significant differences after FDR correction.

Feeding latencies showed that our tests were successful in eliciting novelty and risk responses (Fig. 2): birds started to feed significantly later in the novel object test (paired *t*_57_ = 6.64, P<0.001) and novel food test (paired *t*_57_ = 9.82, P<0.001) than in the first control test; and similarly, latencies in the raptor test were significantly longer than those in the dove test (paired *t*_57_ = 3.86, P<0.001). The changes in latencies throughout the test period indicate that the birds' responses cannot be attributed simply to habituation or cumulative fear effects due to the order of the tests, as they increased or decreased their latencies according to whether or not novelty/risk was present (Fig. 2).

In contrast, the number of hops varied little in response to test situations (control – novel object: paired *t*_57_ = 0.15, P = 0.882; dove – raptor: paired *t*_57_ = 0.40, P = 0.691; Fig. 2) apart from a peak in the novel food test (control – novel food: paired *t*_57_ = 3.63, P = 0.001; Fig. 2). The latter was probably due to the birds' efforts in searching for alternative food sources (note that in the novel food test, the number of hops was not correlated with the latency to feed; Table 2).

**Table 2:** Pairwise Spearman rank-correlations among behavioral variables for all birds (n = 58).

Variable		Latency to feed					Number of hops				
	Test	Control	Object	Food	Dove	Raptor	Control	Object	Food	Dove	Raptor
Latency to feed	Control		0.016*	0.088	0.048	<0.001*	**0.053**	**0.025**	**0.053**	**0.163**	**0.235**
	Object	0.32		0.030	0.926	0.016*	**0.034**	<0.001*	0.003*	0.018*	**0.322**
	Food	0.23	0.29		0.097	0.092	**0.660**	**0.095**	**0.163**	**0.081**	**0.807**
	Dove	0.26	−0.01	0.22		<0.001*	**0.245**	**0.153**	**0.046**	**0.073**	**0.385**
	Raptor	0.49	0.31	0.22	0.44		<0.001*	0.006*	0.004*	0.008*	**0.098**
Number of hops	Control	−**0.26**	−**0.28**	−**0.06**	−**0.16**	−**0.47**		0.001*	0.001*	<0.001*	0.002*
	Object	−**0.29**	−**0.64**	−**0.22**	−**0.19**	−**0.36**	0.44		<0.001*	0.015*	0.141
	Food	−**0.26**	−**0.38**	−**0.19**	−**0.26**	−**0.38**	0.44	0.63		<0.001*	0.042
	Dove	−**0.19**	−**0.31**	−**0.23**	−**0.24**	−**0.34**	0.53	0.32	0.53		<0.001*
	Raptor	−**0.16**	−**0.13**	**0.03**	−**0.12**	−**0.22**	0.40	0.20	0.27	0.63	

Values below the diagonal are correlation coefficients (r_s_). Values above the diagonal are P-values; those marked with asterisk are significant after FDR correction. Correlations between latencies and number of hops are printed in bold.

Individuals' responses were consistent between corresponding situations, as feeding latencies tended to correlate positively between the novel object test and the novel food test (Table 2; P = 0.064 after FDR correction), and correlated significantly between the control test (i.e. the first morning “attack” of the experimenter) and the raptor test (Table 2). This situational consistency implies that latencies in the novelty tests are manifestations of a personality trait related to the exploration-avoidance axis (i.e. neophobia) whereas latencies in the risky situations express another personality trait related to the bold-shy axis (i.e. risk taking). Note that, while all tests involved a short exposure to the experimenter, the sharp decrease in latencies from the control test to the dove test (Fig. 2) suggests that the birds perceived the experimenter more risky first and habituated to her visits later (i.e. latency in the first test might show the birds' fearfulness of humans). The number of hops correlated positively between almost all tests (Table 2), implying that it reflects a third personality trait related to the general activity axis.

### Habitat differences in personality

**Figure 3:**
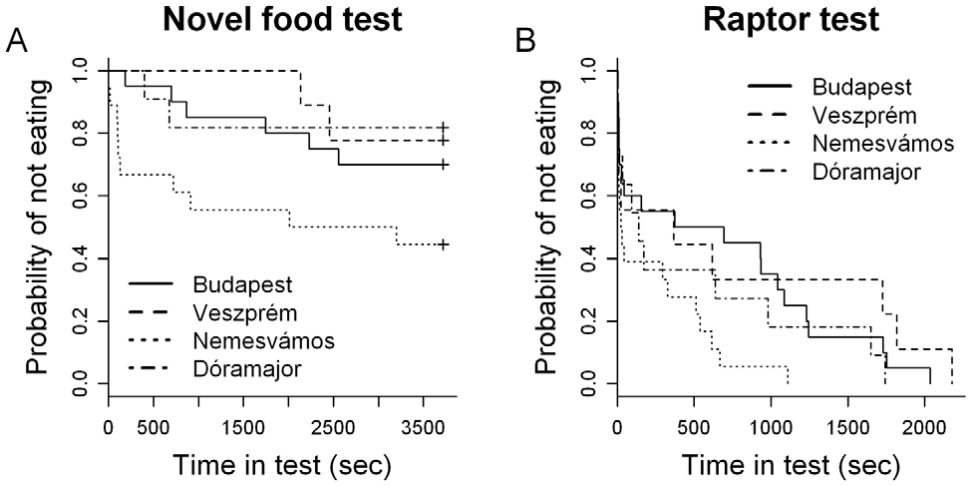
Survival curves showing the probability of not feeding over time for birds of different habitats. Capture sites are listed in order of decreasing urbanization, i.e. from most urbanized Budapest to least urbanized Dóramajor.

Survival analyses showed no significant difference between urban and rural birds in feeding latencies in any of the behavioral tests (Table 3). However, when we compared the four populations, they did not appear that uniform in all tests. First, in the novel food test, we found a marginally non-significant effect of population (Table 3), and post-hoc comparisons indicated that this tendency was due to a significant difference between the two rural populations (Table 4A, Fig. 3). Second, in the raptor test, the effect of population was significant (Table 3), and post-hoc tests indicated that birds from rural Nemesvámos were bolder than the rest of the birds, especially compared to the most and least urbanized populations (although these population differences were not significant after FDR correction; Table 4A, Fig. 3). The number of hops did not vary significantly with habitat or population (Table 5).

**Table 3:** Cox proportional hazards models for latency to feed in the five test situations.

	Urban versusrural			Four populations		
	Deviance	d.f.	P	Deviance	d.f.	P
Control test						
Habitat/Population	1.62	1, 57	0.200	2.23	1, 55	0.530
Novel object test						
Latency to feed in the control test	9.48	1, 57	0.002	9.48	1, 57	0.002
Novel object	12.90	1, 54	0.005	12.90	1, 54	0.005
Habitat/Population	0.02	1, 53	0.876	3.18	3, 51	0.365
Novel food test						
Latency to feed in the control test	5.84	1, 57	0.016	5.84	1, 57	0.016
Novel food	0.49	1, 54	0.921	0.49	1, 54	0.921
Habitat/Population	1.46	1, 53	0.226	7.60	3, 51	0.055
Dove test						
Sex	5.61	1, 57	0.020	5.61	1, 57	0.020
Test group	5.98	1, 56	0.010	5.98	1, 56	0.010
Habitat/Population	1.65	1, 55	0.200	3.67	3, 53	0.300
Raptor test						
Latency to feed in the control test	8.34	1, 57	0.004	8.34	1, 57	0.004
Latency to feed in the dove test	3.19	1, 56	0.070	3.19	1, 56	0.070
Habitat/Population	2.56	1, 55	0.110	8.78	3, 53	0.030

**Table 4:** P-values of pairwise comparisons among four populations for the latencies to feed in five test situations.

	Control	Object	Food	Dove	Raptor
(A) Cox proportional hazards models:					
Budapest-Veszprém	0.951	0.925	0.641	0.194	0.718
Budapest-Nemesvámos	0.273	0.372	0.044	0.212	0.027
Budapest-Dóramajor	0.074	0.478	0.674	0.054	0.811
Veszprém-Nemesvámos	0.981	0.580	0.137	0.991	0.095
Veszprém-Dóramajor	0.156	0.360	0.894	0.369	0.834
Nemesvámos-Dóramajor	0.109	0.114	0.006*	0.579	0.048
(B) Brown-Forsythe tests of variance:					
Budapest-Veszprém	0.892	0.753	0.375	0.973	0.598
Budapest-Nemesvámos	0.385	0.633	0.009*	0.095	0.001*
Budapest-Dóramajor	0.269	0.737	0.792	0.056	0.394
Veszprém-Nemesvámos	0.557	0.484	0.000*	0.282	0.016*
Veszprém-Dóramajor	0.379	0.996	0.585	0.230	0.399
Nemesvámos-Dóramajor	0.702	0.451	0.011*	0.306	0.180

P-values marked with asterisk are significant after FDR correction. The order of capture sites from most to least urbanized is Budapest, Veszprém, Nemesvámos, and Dóramajor.

**Table 5:** Effects of habitat and population on the number of hops.

	*b ±* SE	d.f.	*t*	P
Urban versus rural				
Intercept (Control test, Rural)	3.06±0.18	228	17.14	<0.001
Test type–Novel object	−0.02±0.15	228	−0.16	0.875
Test type–Novel food	0.51±0.13	228	3.86	<0.001
Test type–Dove	0.35±0.14	228	2.55	0.011
Test type–Raptor	0.31±0.14	228	2.25	0.025
Habitat–Urban	−0.10±0.21	49	0.49	0.625
Four populations				
Intercept (Control test, Budapest)	3.11±0.20	228	15.29	<0.001
Test type–Novel object	−0.02±0.15	228	−0.16	0.875
Test type–Novel food	0.51±0.13	228	3.84	<0.001
Test type–Dove	0.35±0.14	228	2.54	0.012
Test type–Raptor	0.31±0.14	228	2.24	0.026
Habitat–Veszprém	−0.14±0.32	47	−0.43	0.672
Habitat–Nemesvámos	0.21 ±0.26	47	0.80	0.428
Habitat–Dóramajor	−0.19±0.30	47	−0.64	0.526

The generalized linear mixed-effects models with quasi-Poisson error distribution included test group and bird ID as nested random factors.

**Figure 4:**
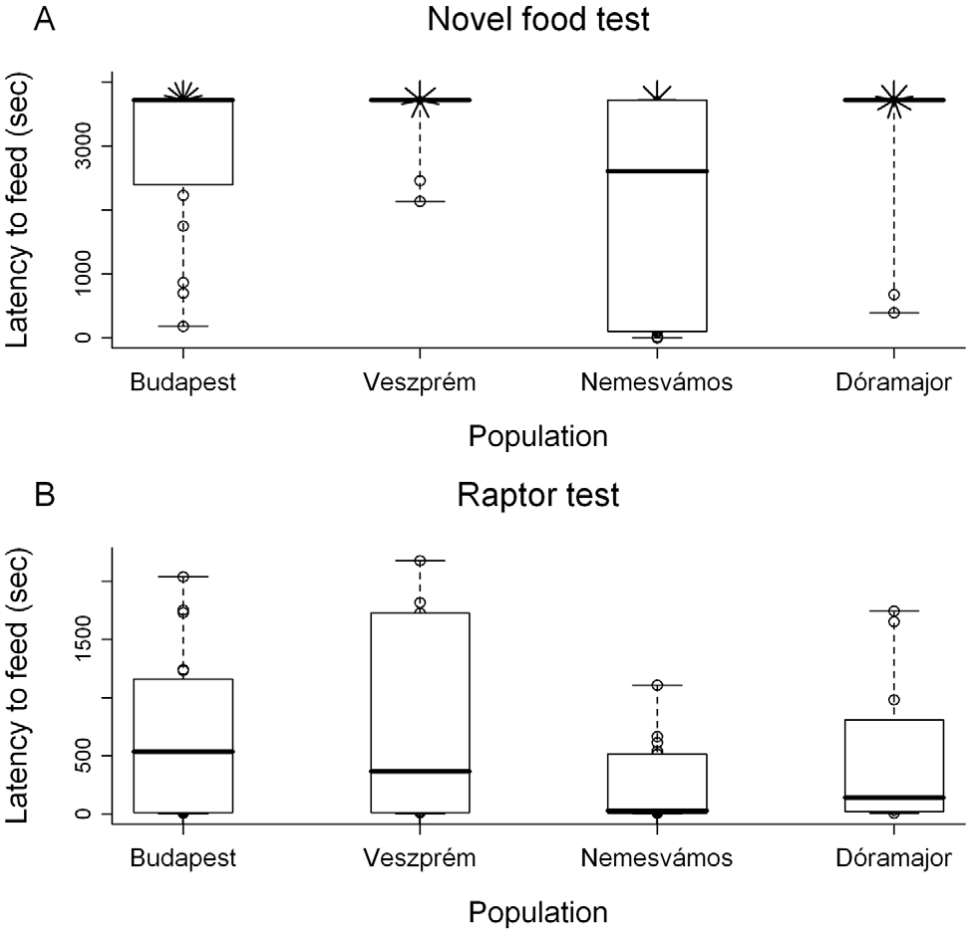
Inter-individual variability of latency to feed in relation to habitat. Box plots show the median (thick line), interquartile range (box) and data range (whiskers) for each habitat; data points for individuals of each capture site are shown as unfilled circles. Overlapping data points are shown as sunflowers; the number of petals correspond to the number of individuals with the same latency value. Capture sites are shown from left to right in order of decreasing urbanization.

Between-individual variance of behavioral traits did not differ between habitats, but we found some differences among populations (Table 6). First, birds from Nemesvámos were more variable than the rest of the birds in the novel food test due to the abundance of very bold individuals (Table 6, Table 4B, Fig. 4). Second, an opposite trend appeared in the raptor test, i.e. the Nemesvámos population was less variable than the two more urbanized populations due to the significant scarcity of very shy individuals in the former (Table 6, Table 4B, Fig. 4).

**Table 6:** Brown–Forsythe tests comparing the variance of behavioral variables among birds from different habitats and populations.

		Urban versus rural		Four populations	
Variable	Test	*F* (d.f. = 1,57)	P	*F* (d.f. = 3,54)	P
Latency to feed	Control	1.59	0.212	0.57	0.635
	Object	0.39	0.535	0.22	0.879
	Food	3.42	0.070	4.44	0.007*
	Dove	4.35	0.041	1.58	0.205
	Raptor	5.96	0.018	3.16	0.032
Number of hops	Control	0.03	0.859	0.49	0.691
	Object	0.33	0.570	0.42	0.741
	Food	1.81	0.185	0.67	0.575
	Dove	1.72	0.195	0.56	0.647
	Raptor	1.34	0.252	0.61	0.612

P-values marked with asterisk are significant after FDR correction.

### Behavioral syndromes

**Figure 5:**
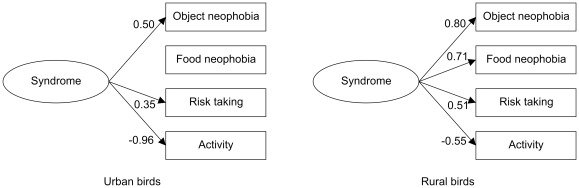
The most supported structural equation model of syndrome structure for urban and rural birds. Numbers associated with the arrows are standardized effects of the underlying syndrome structure on behavioral traits.

Overall, the correlations among several variables expressing neophobia, risk taking and activity (Table 2) suggest a syndrome structure in house sparrows. SEM analyses clearly supported the existence of a behavioral syndrome for both urban and rural birds, as the model assuming no syndrome had relatively high DIC (ΔDIC>9) in both groups (Table 7). However, the most supported model of syndrome structure (ΔDIC<3) was different for urban and rural birds, the urban syndrome structure showing more restricted domain-generality than the rural syndrome structure (Table 7). Specifically, the most supported model for urban birds indicated that food neophobia was not part of their behavioral syndrome (Fig. 5), whereas the most supported model for rural birds showed that all four personality traits were strongly integrated into their syndrome structure (Fig. 5).

**Table 7:** Comparison of Bayesian structural equation models of syndrome structure for urban and rural birds.

	Urban		Rural	
	DIC	ΔDIC	DIC	ΔDIC
Model 1	146.75	3.20	**137.65**	**0**
Model 2	**143.55**	**0**	147.33	9.68
Model 3	153.13	9.58	141.73	4.08
Model 4	152.92	9.37	153.02	15.37

Models are evaluated based on differences in deviance information criterion (DIC) values calculated from Markov chain Monte Carlo simulations. For each group, the most supported model (i.e. the one with the lowest DIC value) is highlighted in bold. See Figure 1 for explanation of Models 1–4.

## Discussion

We have found individual consistency in three behavioral traits in house sparrows, which correspond to three main dimensions of animal personality, namely exploration-avoidance, shyness-boldness, and activity [18]. We have also found evidence that these personality traits form a behavioral syndrome. These results complement a recent study [38] that found in rural male sparrows that both activity and exploration of a novel environment were repeatable over time but did not correlate with each other.

We did not find consistent differences between urban and rural birds either in the averages or the variances of personality traits, but the four study populations were not entirely similar in these aspects. Most differences appeared between one rural site with largest sample size and some or the rest of the other populations. These results can be interpreted in several alternative ways. Firstly, the effects of urbanization on behavioral traits may be non-linear, e.g. selection for certain personalities might be strongest at medium levels of habitat urbanization (Nemesvámos may represent this in our sample). This is an intriguing possibility given that urbanization is known to have non-linear or threshold effects on biological phenomena such as population densities [3], [49] and behavior, including the house sparrows' responses to human disturbance [50]. Secondly, behavioral traits may vary linearly along the urban gradient, and the discrepancy between our two rural sites in some variables might be due to sampling error. The relatively small sample from our least urbanized population might not be representative enough for the rural end of the urban gradient; for example, birds captured from Dóramajor had lower weight than that expected by the low urbanization of their habitat [35]. Finally, behavioral differences might also be related to variation in some other habitat characteristics across populations which is only weakly or not at all related to urbanization. For instance, environmental fluctuations in food distribution or predation risk independent of urbanization may exert selection pressure on personality traits [17], [22], [24]. Further studies sampling a wider variety of populations along the urban gradient are needed to evaluate these alternatives.

Response to novelty is one of the personality traits most likely to be involved in animals' adaptation to “city life”, given that reduced fear of novel stimuli and adopting novel behaviors is known to be adaptive during invasion of novel environments [28], [41], [51]. Despite this expectation, our study yielded no evidence for reduced neophobia in urban birds. On the contrary, we found that birds from the second least urbanized habitat were the most willing to taste novel food items, whereas responses to novel objects did not differ across populations. These findings are consistent with the results of previous studies which suggested that fear of novel objects is not reduced or even increased in urban house sparrows compared to less urbanized conspecifics [26]–[27], [32]. The dangerous niche hypothesis [52] proposes that increased wariness of novelty is adaptive when novel stimuli are often dangerous (e.g. traps, poisons), therefore species living under such conditions are best off combining exploration with initially high neophobia when encountering unfamiliar things. Thus, while reduced neophobia may be adaptive during the colonization of cities [28], [41], persisting there might necessitate more cautious behavior towards novelty [26]. This idea is open for further investigations, predicting for example that neophobia should first decrease while getting urbanized but increase after the population has settled in its new urban habitat, similarly to the dynamics of flight initiation distances during birds' urbanization [10]–[11].

Response to predation risk is another personality trait that may play central role in adaptation to urban environments, as suggested by the numerous studies showing that urbanization, or more particularly the high densities of humans, reduce animals' fearfulness of humans [7]–[8], [10]–[11], [53]. However, humans are a very special type of predator as they rarely hunt and eat urban animals, thus their presence can be habituated to whereas repeated exposure to real predators tends to increase the fearfulness of prey 32,53. The density of non-human predators changes in a complex manner along the gradient of urbanization [16], and human disturbance may further complicate the anti-predatory responses of prey populations [31]. Here we have found that sparrows from the second least urbanized habitat tended to be least fearful after being exposed to the sparrowhawk dummy, as very few of them were extremely shy. This finding can be interpreted in light of the result of another study on a different set of sparrows from ten populations showing that, among non-naive sparrows, urban birds are more fearful of the sparrowhawk than rural birds [32]. This may be adaptive if urban birds are more exposed to predation risk, which is a sound possibility regarding the house sparrow and the sparrowhawk [32]. Interestingly, however, activity which was correlated with risk-taking and is often considered a risky trait [19], [54] did not differ between birds from different habitats in our study. Our inability to detect robust habitat differences in both risk-taking and activity might be due to the fact that, in contrast to [32], we could not assess the age of the birds in the present study.

Recently, it has been proposed that urbanization may select not only for certain behavioral traits but also for inter-individual variability of those traits as a manifestation of behavioral flexibility [8], [11]. Comparative studies of avian flight initiation distances suggested that urban habitats are colonized by a relatively homogenous subset of individuals of variable species [8], [11], then inter-individual variability increases as the population develops more diversified ways of exploiting the urban environment [11]. Because population densities in cities often exceed those in natural habitats, strong competition may intensify the selection for diverse personalities to the extent that urban populations become more diverse than rural ones in species with a long history of urbanization [11]. Our results partially support this scenario, as we have found differences in variance among differently urbanized populations in fearfulness of raptors and food neophobia, but the direction of these differences were inconsistent both along the urban gradient and between the two variables, and the rest of the behavioral traits in our study were similarly variable in all habitats. These results may suggest that urbanization has differential effects on the inter-individual variability of different behaviors. Further studies are needed to ascertain when and why urbanization selects for an increase in behavioral flexibility, in terms of both inter-individual variability and intra-individual flexibility.

Finally, we have found that response to novelty, risk taking and activity form a behavioral syndrome in house sparrows. SEM analyses indicated that syndrome structure is not the same in urban and rural birds, implying that different combinations of personality traits may be adaptive in different habitats. The major difference between urban and rural sparrows was that food neophobia was part of the behavioral syndrome in the latter but not in the former, suggesting that perhaps urban birds evolved more diversified ways of exploiting food resources than rural birds. Interestingly, studies on fish found that behavioral syndromes break down under favorable conditions such as in habitats with low predation [23]–[24] or high-quality breeding sites [55], and studies on song sparrows found a similar breakdown in urban populations [33]–[34]. In contrast, in house sparrows the behavioral syndrome did not break down in urban birds, although its domain-generality became more restricted compared to rural birds. This is in line with recent findings indicating that urban and rural areas may represent habitats of similar quality overall for non-breeding house sparrows [35], [56] even though they may differ in specific components of habitat quality such as predation risk [32].

In sum, our study has demonstrated consistent individual differences in three personality traits of house sparrows, some differences in the mean and variance of these traits across populations, and an urban-rural habitat difference in syndrome structure. Although alternative explanations cannot be discarded, these results altogether imply that urbanization may influence the distribution and diversity of individually consistent behaviors, selecting for populations with altered personality, behavioral flexibility, and syndrome structure. Similar studies on species at different stages of urbanization would help to advance our understanding on the dynamics of animals' behavioral adapatation to urban life.
